# The uniqueness of subjective ageing: convergent and discriminant validity

**DOI:** 10.1007/s10433-019-00529-7

**Published:** 2019-10-09

**Authors:** Svenja M. Spuling, Verena Klusmann, Catherine E. Bowen, Anna E. Kornadt, Eva-Marie Kessler

**Affiliations:** 1 German Centre of Gerontology (DZA), Manfred-von-Richthofen-Str. 2, 12101 Berlin, Germany; 2 Department of Psychology, Psychological Assessment and Health Psychology, University of Konstanz, P.O. Box 47, 78457 Constance, Germany; 3 Department of Psychology and Human Movement Science, Public Health, University of Hamburg, Mollerstr. 10, 20148 Hamburg, Germany; 4 Vienna, Austria; 5 Fakultät für Psychologie und Sportwissenschaft, Differentielle Psychologie und Psychologische Diagnostik, Bielefeld University, Universitätsstraße 25, 33615 Bielefeld, Germany; 6 Department of Psychology, Geropsychology, MSB Medical School Berlin, Calandrellistraße 1-9, 12447 Berlin, Germany

**Keywords:** Subjective ageing, Subjective age, Self-perceptions of ageing, Attitude toward own ageing, Ageing cognitions, Validity

## Abstract

Although a large body of research has demonstrated the predictive power of subjective ageing for several decisive developmental outcomes, there remains some controversy about whether subjective ageing truly represents a unique construct. Thus, information about the convergent and discriminant validity of different approaches to measuring subjective ageing is still critically needed. Using data from the 2014 wave of the German Ageing Survey, we examined how three established subjective ageing measures (subjective age, global attitude toward own ageing, multidimensional ageing-related cognitions) were inter-related as well as distinct from general dispositions (optimism, self-efficacy) and well-being (negative affect, depressive symptoms, self-rated health). Using correlational and multivariate regression analysis, we found that the three subjective ageing measures were significantly inter-related (*r* = |.09| to |.30|), and that each measure was distinct from general dispositions and well-being. The overlap with dispositional and well-being measures was lowest for subjective age and highest for global attitudes towards own ageing. The correlation between global attitudes towards own ageing and optimism was particularly striking. Despite the high convergent validity of the different dimensions of ageing cognitions, we nevertheless observed stronger associations between specific dimensions of ageing cognitions with negative affect and self-rated health. We conclude that researchers should be aware of the multidimensional nature of subjective ageing. Furthermore, subjective age appears to be a highly aggregated construct and future work is needed to clarify its correlates and reference points.

**Electronic supplementary material:**

The online version of this article (10.1007/s10433-019-00529-7) contains supplementary material, which is available to authorized users.

## Introduction

Research on subjective ageing—that is, personal representations of one’s own old age and the ageing process—has surged over the last decade. By now, a plethora of studies have demonstrated that subjective ageing is a powerful predictor of many important outcomes (for a review, see, e.g. Kornadt et al. 2019). Nevertheless, there remains some controversy about whether subjective ageing truly represents a unique construct, and to date no study has systematically examined the convergent and discriminant validity of different subjective ageing measures. We therefore use representative data from adults 40 + in Germany to determine the extent to which three widely used subjective ageing measures are in fact empirically similar and/or distinct from one another as well as five more general dispositional (optimism, self-efficacy) and well-being variables (negative affect, depressive symptoms, self-rated health).

### Three key measures of subjective ageing: subjective age, attitudes toward own ageing subscale and the ageing cognitions scales

Three measures most commonly used within the subjective ageing literature (cf. Westerhof and Wurm 2015) are the single-item assessment of *subjective age* (SA, Kastenbaum et al. 1972; Montepare and Lachman 1989), the *Attitudes Toward Own Ageing* (ATOA) subscale of the Philadelphia Geriatric Morale Scale (PGCMS, Lawton 1975) and the multidimensional *Ageing Cognitions Scales* (*AgeCog,* Steverink et al. 2001). SA assesses how old people feel in relation to their chronological age. ATOA assesses people’s overall, global attitude towards their own ageing along a single positive–negative dimension. Finally, the AgeCog scales capture how individuals feel about how they have changed and/or will change as they get older in four distinct dimensions, namely physical decline (e.g. loss in health or vitality), social losses (e.g. no longer being needed by others or decreased respect), continuous growth and personal development, and self-knowledge (Steverink et al. 2001; Wurm et al. 2007; Klusmann et al. 2019b). Researchers have also recently used the three measures to validate newer subjective ageing constructs (Brothers et al. 2017).

Research has already convincingly demonstrated that responses to SA, ATOA and AgeCog are each meaningfully related to a number of important outcomes, both cross-sectionally and longitudinally. For instance, people who report a younger SA generally indicate better psychological well-being, health and cognitive functioning and even lower mortality (for recent examples, see Kotter-Grühn et al. 2009; Stephan et al. 2011, 2013). Similarly, several studies have demonstrated that scores on the ATOA predict physical and cognitive functioning or mortality over and above, for example, self-efficacy, self-rated health (Siebert et al. 2018; Tovel et al. 2019) or hope (Levy et al. 2002). Scores on the AgeCog scales have been found to be longitudinally related to health over and above control beliefs (Wurm et al. 2007) and to physical exercise (Wurm et al. 2010). More recently, Klusmann and colleagues (2019b) showed that scores on the AgeCog self-knowledge scale predicted favourable changes in eating behaviour. Wolff and colleagues (2017) showed that responses to the AgeCog physical losses scale were linked to affect 6 months after a serious health event and to functional limitations 2.5 years later.

The predictive power of the SA, ATOA and AgeCog measures is convincing. Despite some evidence to the contrary, however, there remains some controversy about whether “subjective ageing” truly represents a unique construct distinct from people’s general tendency to see things in a positive/negative light or their well-being (cf. Gendron et al. 2018; Jung and Siedlecki 2018; Wurm et al. 2007). Furthermore, to date there has been little stringent theoretical elaboration regarding the extent to which the SA, ATOA and AgeCog measures tap into the same or different aspects of subjective ageing (though see Diehl et al. 2014 for a recent exception), nor has any study empirically examined the extent to which the three measures actually converge (i.e. are related, which should be the case if “subjective ageing” truly exists).

### The need to examine the construct validity of different subjective ageing measures

Information about the construct validity of the SA, ATOA and AgeCog measures is an important pre-requisite for being able to appropriately interpret and integrate subjective ageing research. Without knowing the extent to which different subjective ageing measures converge, for instance, it is unclear whether subjective ageing as an umbrella construct in fact exists, or whether it in fact makes sense to treat the three different measures as if they measure the same thing. One notable conceptual difference between the measures is that SA is measured using a scale of years, thus anchoring it to a person’s chronological age. In contrast, ATOA and the AgeCog scales are rated on arbitrary Likert-type scales. In addition, SA and ATOA assess global evaluations, while the AgeCog scales capture dimension-specific evaluations (e.g. physical losses, social losses). The scales also obviously differ with regard to the number of items and whether items refer to the past, present, and/or future (see Methods section). Nevertheless, in research articles, authors have often summarized the state of the literature as if the three measures were interchangeable, and a number of recent meta-analyses and qualitative reviews have integrated and summarized studies using the three measures as well (e.g. Westerhof and Wurm 2015; Westerhof et al. 2014; Wurm et al. 2017).

Without information on how different subjective ageing measures are inter-related, it is also unclear whether results based on one measure are likely to generalize to other subjective ageing measures (e.g. whether evidence that casts doubt on the content or construct validity of SA extends to the whole subjective ageing construct). The ability to generalize across measures is particularly important when it comes to making hypotheses about working mechanisms or designing interventions. If, for instance, scores on SA, ATOA and AgeCog are highly related, then it can be presumed that interventions that successfully affect scores on one measure (e.g. feedback about one’s handgrip strength relative to age peers which affects response to SA; Stephan et al. 2013) might also affect how people respond to the other measures (e.g. change how people respond to items about age-related physical losses or ongoing development).

### Existing evidence on the uniqueness of subjective ageing

Previous research has provided at least some evidence about the discriminant validity of each measure vis-à-vis more general dispositional and well-being variables. For example, using a representative sample of German adults 40 + years, Jung and Siedlecki (2018) demonstrated that ATOA can be discriminated from overall life satisfaction as well as positive and negative affect. AgeCog retains predictive power even after controlling for other more general dispositional and well-being variables such as hope and loneliness (Steverink et al. 2001) and control beliefs (Wurm et al. 2007). While some recent studies demonstrate the predictive validity of SA over and above more general dispositional and well-being indicators (e.g. Stephan et al. 2018b; Rippon and Steptoe 2015), other studies have found that SA has no predictive power once more general indicators have been statistically controlled (e.g. Zacher and Rudolph 2018). Gendron and colleagues (2018) recently presented persuasive arguments that cast doubt on the content and construct validity of the single-item SA measure. In sum, existing evidence does support the idea that different subjective ageing measures capture something unique from general dispositions and well-being, though evidence on the whole is still rather limited.

### The present study

Many studies have convincingly demonstrated that responses to the SA, ATOA and AgeCog measures are each meaningfully associated with a number of important outcomes, both cross-sectionally and longitudinally. Information about the convergent and discriminant validity of different subjective ageing measures is, however, still critically needed. In the current study, we therefore used representative data from older German adults (40 + years) to examine the relationships between responses to SA, ATOA and AgeCog, first, with each other and, second, with two dispositional variables (optimism, self-efficacy) and three well-being variables (negative affect, depressive symptoms and self-rated health). Our goals were to demonstrate the uniqueness of the subjective ageing construct as a whole as well as to compare the convergent and discriminant validity of each separate measure.

We expected that responses to SA, ATOA and AgeCog would be significantly correlated with each other and that each measure would be empirically distinct from the general dispositional and well-being variables. However, because SA, ATOA and AgeCog measures assess conceptually different phenomena, we also expected that the intercorrelations between the three measures would be only moderate in magnitude, and that the degree to which each measure was distinct from the general dispositional and well-being variables would vary.

## Method

### Sample

Data came from the German Ageing Survey (DEAS), an ongoing nationwide representative cohort-sequential survey of the German community-dwelling population aged 40 years and older. Starting in 1996, every 6 years a new baseline sample has been drawn by means of national probability sampling, being systematically stratified by age, gender and region (former West or East Germany). The present study uses cross-sectional data from the most recent baseline sample of 2014 (*N* = 6002). Participants of the DEAS attend a personal computer-assisted interview and are asked to complete an additional self-administered questionnaire. Only those participants who completed the self-administered questionnaire were included in the present study (*n* = 4295), since most of the study variables were assessed in the questionnaire only. Women, older participants and participants with a higher socio-economic status (better education and higher income) were more likely to complete the additional questionnaire (Klaus and Engstler 2017). However, the magnitude of these selection effects was rather low.

### Measures

#### Subjective ageing measures

Participants were asked about their felt age: “Forget your actual age for a moment: How old do you feel, if you had to express it in years?” *SA* was measured as the difference between felt age and chronological age divided through chronological age resulting in a proportion score that represents how much younger/older participants felt relative to their chronological age. Negative values indicate feeling younger than one’s chronological age, while positive values indicate feeling older. Higher values indicate a larger discrepancy between felt and chronological age. Extreme outliers (three standard deviations below or above the sample mean) were deleted (*n* = 20).

Global attitudes towards own ageing was measured with the *ATOA* subscale from Lawton’s (1975) PGCMS. Participants were asked to indicate the extent to which they agreed with five statements (e.g. “Things keep getting worse as I get older”) on a four-point scale ranging from 1 (strongly agree) to 4 (strongly disagree). Scores were averaged and higher values indicate a more positive global attitude towards own ageing.

Finally, multidimensional positive and negative ageing-related cognitions were measured with the *AgeCog scales* (Klusmann et al. 2019b; Steverink et al. 2001; Wurm et al. 2007). Participants indicated the extent to which different statements reflected their own views on ageing (“Ageing means to me that…”) regarding physical losses (“…I am less energetic and fit”), social losses (“…I feel less needed), continuous growth and ongoing development (“…I can still learn new things”), and increased self-knowledge (“…I know myself better”). Each scale consists of four statements rated using a 4-point scale ranging from 1 (strongly agree) to 4 (strongly disagree). For each scale, scores were averaged and higher values indicate either more negative ageing cognitions (AgeCog Physical Losses and AgeCog Social Losses) or more positive ageing cognitions (AgeCog Ongoing Development and AgeCog Self-Knowledge).

#### General dispositional measures

*Optimism* regarding one’s future was measured with the Affective Valence of the Future Time Perspective Scale (Brandtstädter and Wentura 1994). Participants indicated the extent to which they agreed with five statements (e.g. “For me the future is full of hope”) on a four-point scale from 1 (strongly agree) to 4 (strongly disagree). *Self*-*efficacy* was measured with the General Self-Efficacy scale (Schwarzer and Jerusalem 1995). Participants indicated the extent to which they agreed with five statements (e.g. “I can usually handle whatever comes in my way”) on a four-point scale ranging from 1 (strongly agree) to 4 (strongly disagree). Both scales were recoded so that higher values indicate more optimism and more self-efficacy, respectively.

#### Well-being measures

*Depressive symptoms* were assessed with the German version of the 15-item CES-D scale (Center for Epidemiological Studies Depression scale; Hautzinger 1988). Participants indicated the frequency of several depressive symptoms (e.g. being sad, trouble sleeping) during the past week using a four-point scale ranging from 1 (rarely or none of the time) to 4 (most or all of the time). A sum score between 0 and 45 was computed, with higher values indicating more frequent depressive symptoms. *Negative affect* was measured with the PANAS (Positive and Negative Affect Schedule; Watson et al. 1988). Participants rated the intensity of 10 negative affective states (e.g. distressed, nervous) during the past few months using a five-point scale ranging from 1 (very slightly or not at all) to 5 (extremely). Higher values indicate more negative affect. *Self*-*rated health* was measured with a single item: “How would you rate your present state of health?” answered on a 5-point scale ranging from 1 (very good) to 5 (very bad). The item was recoded, so that higher values indicate better self-rated health.

#### Control variables

The DEAS is disproportionately stratified by age, gender and region (East/West Germany); hence, these three variables were used as statistical controls. The inclusion of the sample stratification factors as covariates in the models nullifies the need for sample weights (Winship and Radbill 1994). Education (three categories according to the International Standard Classification of Education; United Nations Educational, Scientific and Cultural Organization 2011) was included as an additional control variable.

### Statistical analysis

We followed a four-step analytic strategy. First, we examined the descriptive characteristics and scale reliabilities. The maximum correlation between two variables is equal to the square root of the product of the two scale reliabilities (Howitt and Cramer 2005; p. 166). Second, we examined the intercorrelations among SA, ATOA, the AgeCog scales as well as with the general dispositional (optimism, self-efficacy) and well-being variables (depressive symptoms, negative affect, self-rated health) based on both raw correlations as well as the correlations corrected for scale reliability (i.e. the raw correlation divided by the respective maximum correlation; Howitt and Cramer 2005).

Third, we used multiple regression analysis to assess how much variance (*R*^2^) in each subjective ageing measure could be explained by the dispositional, well-being and control variables. We ran separate models for each subjective ageing measure, first adding the control variables (age, gender, region, education) and then the general dispositional and well-being measures blockwise. Finally, we conducted commonality analyses to assess how much unique variance in SA, ATOA and AgeCog was explained by each of the dispositional and well-being variables. Specifically, we ran a series of regression analyses in which each dispositional and well-being variable was entered last to a model with all of the other dispositional and well-being variables already included as predictors. The change in *R*^2^ indicates the amount of variance in subjective ageing exclusively attributed to the last-entered variable, over and above the other predictors.

Missing data was very low (< 2.6% across all study items) and was listwise deleted. The alpha level was set to 5%. However, given the size of the sample, we interpret significance levels exceeding *p* > .01 with some caution. We consider significance levels as well as the magnitude of the coefficients when interpreting the results of the regression analyses.

## Results

The average age of respondents was 62.33 years (age range 40–85 years; SD = 11.47), 50.4% of the sample were female, 32.2% were living in former East Germany, and 6.9% had a low level of education (i.e. nine or fewer years of education). Table 1 shows descriptive characteristics for the subjective ageing, general dispositional and well-being variables.

**Table 1:** Sample characteristics for the total sample (N = 4295)

Measures [sample range]	Means (SD)
Subjective ageing measures	
SA [− .66–.41]	− .13 (.12)
ATOA [1–4]	2.96 (.56)
AgeCog	
Physical losses [1–4]	2.77 (.53)
Social losses [1–4]	1.84 (.55)
Ongoing development [1–4]	2.91 (.56)
Self-knowledge [1–4]	3.03 (.46)
General dispositional measures	
Optimism [1–4]	2.99 (.55)
Self-efficacy [1–4]	3.08 (.44)
Well-being measures	
Depressive symptoms [0–44]	6.89 (6.11)
Negative affect [1–5]	2.12 (.53)
Self-rated health [1–5]	3.50 (.85)

*SA* subjective age, *ATOA* attitude toward own ageing subscale, *AgeCog* ageing cognitions scales

### Relationships among subjective ageing measures

Table 2 displays the zero-order correlations among the subjective ageing, general dispositional and well-being measures, based on the raw correlations. The correlations corrected for scale reliability were also calculated and patterns of correlations were similar (see Supplementary Appendix). The relationships between ATOA and the AgeCog scales, range abs(*r*) = .35 to .61, were considerably higher than either the correlation between SA and ATOA, *r* = − .30, or the correlations between SA and the AgeCog scales, range abs(*r*) = .09 to .23.

**Table 2:** Bivariate correlations between subjective ageing measures and general dispositional and well-being variables for the total sample

	Cronbach’s alpha	1	2	3	4	5	6	7	8	9	10
1 SA											
2 ATOA	.74	− .30									
3 AgeCog physical losses	.76	.23	− .53								
4 AgeCog social losses	.71	.17	− .61	.44							
5 AgeCog ongoing development	.78	− .22	.60	− .40	− .43						
6 AgeCog self-knowledge	.57	− .09	.35	− .11	− .26	.42					
7 Optimism	.83	− .23	.69	− .44	− .51	.61	.37				
8 Self-efficacy	.74	− .18	.47	− .29	− .38	.52	.42	.57			
9 Depressive symptoms	.86	.21	− .45	.28	.36	− .28	− .15	− .44	− .32		
10 Negative affect	.85	.12	− .37	.24	.41	− .20	− .19	− .40	− .39	.48	
11 Self-rated health		− .27	.45	− .40	− .24	.33	.14	.38	.26	− .46	− .23

All coefficients significant at *p* < .01

*SA* subjective age, *ATOA* attitude toward own ageing subscale, *AgeCog* ageing cognitions scales

### Relationships between subjective ageing, general dispositional and well-being measures

Of the different subjective ageing measures, the general dispositional and well-being variables were least strongly associated with SA, range abs(*r*) = .12 to .27, and most strongly associated with ATOA, range abs(*r*) = .37 to .69.

Among the different general dispositional or well-being variables, optimism was the most highly correlated with the subjective ageing measures, range abs(*r*) = .23 to .69. The high correlation between optimism and ATOA is particularly noteworthy, *r* = .69, as well as the high variation in the correlations between the general dispositional and well-being variables and the different AgeCog scales. For instance, self-rated health was primarily related to AgeCog Physical Losses (*r* = − .40) while negative affect was primarily related to AgeCog Social Losses (*r* = .41). Self-efficacy, in contrast, was primarily associated with cognitions about ageing-related developmental gains, that is, with AgeCog Ongoing Development (*r* = .52) and, to a somewhat lesser extent, AgeCog Self-Knowledge (*r* = .42).

### Variance in subjective ageing measures explained by general dispositional and well-being measures

Table 3 displays the results of the multiple regression analyses; relationships with a significance level of *p* < .01 are in bold. The general dispositional, well-being and control variables explained a significant amount of variance for each subjective ageing measure. The amount of explained variance ranged from 9.7% for SA to 54.9% for ATOA. Total variance explained by the predictors altogether ranged from 46.0% in AgeCog Ongoing Development, to 32.4% in AgeCog Social Losses, to 26.3% in AgeCog Physical Losses, to finally only 21.5% in AgeCog Self-Knowledge.

**Table 3:** Standardized regression coefficients and determination coefficients from multiple regression analysis of subjective ageing measures

	SA	ATOA	AgeCog physical losses	AgeCog social losses	AgeCog ongoing development	AgeCog self-knowledge
Predictor variables						
Optimism	− **.11**	**.51**	− **.28**	− **.35**	**.44**	**.23**
Self-efficacy	− *.05*	**.07**	− *.04*	− **.07**	**.27**	**.31**
Depressive symptoms	**.08**	− **.09**	.00	**.08**	.01	*.04*
Negative affect	− .01	− **.08**	**.08**	**.21**	**.07**	.02
Self-rated health	− **.17**	**.16**	− **.25**	.01	**.08**	.02
Controls						
Age	.00	− **.05**	**.09**	.02	− **.12**	**.12**
Gender	− **.07**	**.07**	− *.03*	− **.04**	.02	**.07**
Region	*.04*	− **.05**	− .00	− .00	− *.03*	.02
Education	− .02	**.07**	.01	− **.06**	**.10**	− .02
Determination coefficient (*R*^2^)	.097	.549	.263	.324	.460	.215
Sample size	4080	4110	4098	4071	4065	4095

Displayed are the standardized coefficients (β) from separate stepwise regression models for each subjective ageing measure as the dependent variable, based on the total sample. Significant coefficients at *p* < .01 are printed in bold. Significant coefficients at *p* < .05 are printed in italic

*SA* subjective age, *ATOA* attitude toward own ageing subscale, *AgeCog* Ageing cognitions scales

Both dispositional measures (optimism and self-efficacy) were significantly associated with every subjective ageing measure in the multiple regression models. Depressive symptoms were neither significantly related to AgeCog Physical Losses nor to AgeCog Ongoing Development over and above the other predictors. Similarly, negative affect was not significantly associated with SA, and self-rated health was not significantly associated with AgeCog Social Losses. Finally, neither negative affect nor self-rated health was significantly associated with AgeCog Self-Knowledge.

**Fig. 1:**
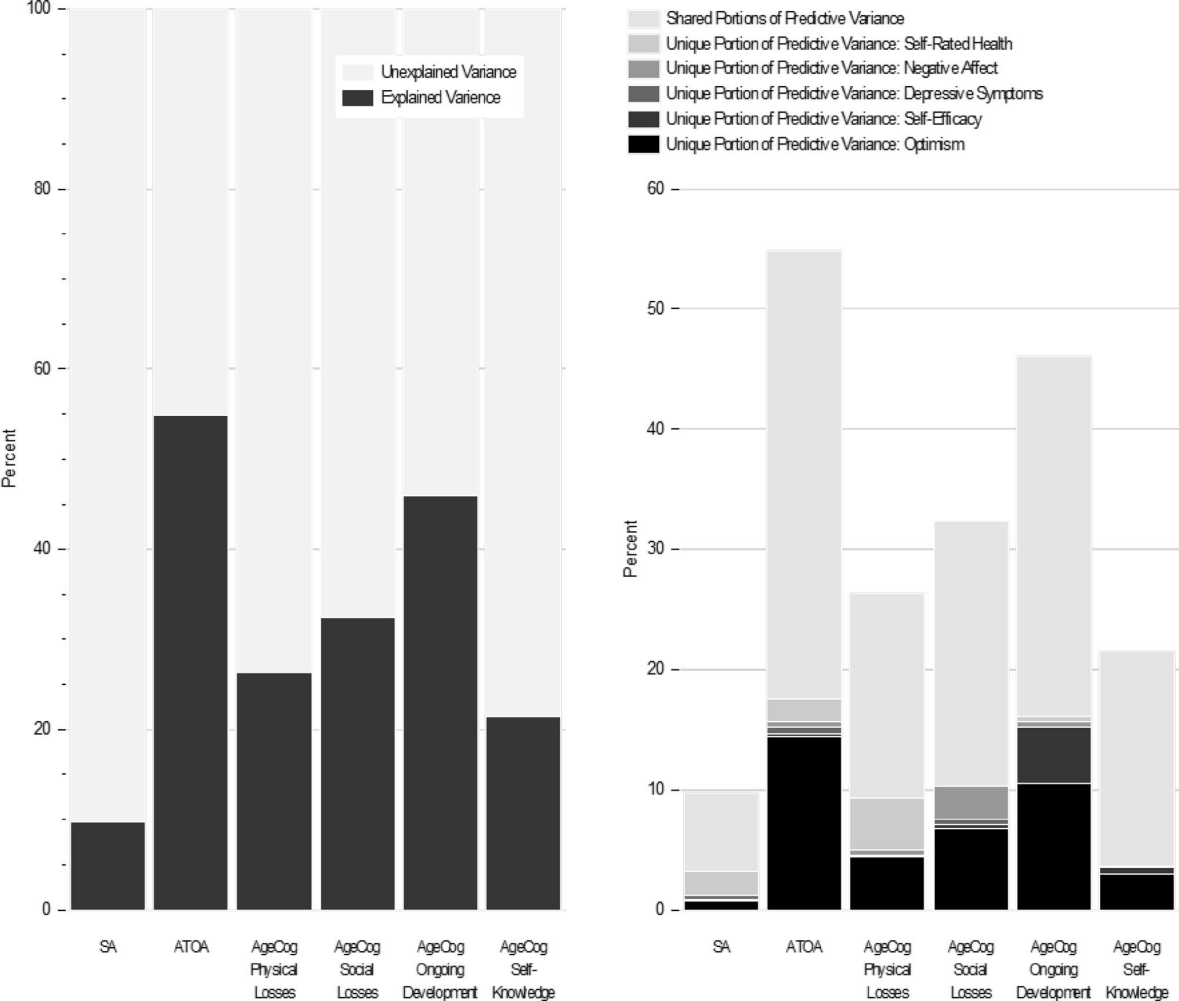
Commonality analysis: unique, shared, and unexplained portions of variance of the studied subjective ageing measures. Ratio of unexplained and explained variance (left); unique proportion of predictive variance for each predictor (right). *SA* subjective age, *ATOA* attitude toward own ageing subscale, *AgeCog* ageing cognitions scales

Table 4 displays the results of the commonality analyses. The proportion of variance explained by the general dispositional and well-being measures all together ranged from 6.5% for SA to 37.4% for ATOA. Optimism uniquely explained the largest—though still modest—share of variance for ATOA, AgeCog Physical Losses (along with self-rated health), AgeCog Social Losses and AgeCog Self-Knowledge (range: 2.9% for AgeCog Self-Knowledge to 14.4% for ATOA). Self-rated health explained the greatest share of unique variance in SA (2.0%) and AgeCog Physical Losses (4.4%). Self-efficacy only uniquely explained a substantial proportion of variance in AgeCog Ongoing Development (4.7%), and negative affect only uniquely explained a substantial proportion of variance in AgeCog Social Losses (2.8%). Figure 1 displays the proportion of unexplained variance for each subjective ageing measure as well as the proportion variance explained by the general dispositional and well-being measures all together and by each measure uniquely.

**Table 4:** Commonality analysis: percent variance in subjective ageing measures explained by general dispositional and well-being variables

Variance (%)	SA	ATOA	AgeCog physical losses	AgeCog social losses	AgeCog ongoing development	AgeCog self-knowledge
Unique						
Optimism	.7	14.4	4.4	6.8	1.5	2.9
Self-efficacy	.1	.3	.1	.3	4.7	.6
Depressive	.4	.5	0^a^	.4	0^a^	.1
Negative affect	0^a^	.4	.4	2.8	.4	0^a^
Self-rated health	2.0	1.9	4.4	0^a^	.5	0^a^
Shared	6.5	37.4	17.0	22.1	29.9	17.9
Total explained (%)	9.7	54.9	26.3	32.4	46.0	21.5
Unexplained (%)	93.3	45.1	73.7	67.6	54.0	78.5

Percent unique, shared, and unexplained variance from separate regression models with each subjective ageing measure as the dependent variable, based on the total sample. All models are controlled for age, gender, region, and education

*SA* subjective age, *ATOA* attitude toward own ageing subscale, *AgeCog* ageing cognitions scales

^a^Non-significant change in *R*^2^ (meaning non-significant unique portion of predictive variance)

## Discussion

Using representative data from the German Ageing Survey (DEAS), we provide evidence on the convergent and divergent validity of three established measures of subjective ageing, namely SA, the ATOA subscale of the PGCMS, and the four AgeCog scales. Although it has been shown that each of these measures is linked to important developmental and health outcomes, until now no study had systematically examined the extent to which these measures tap into the same underlying construct. Also, there was a need to demonstrate that subjective ageing is something other than a reflection of people’s general dispositions and well-being.

Most importantly, our findings clearly demonstrate the uniqueness of the subjective ageing construct, specifically, that subjective ageing is *not* just another expression of a person’s optimism, self-efficacy, depressive symptoms, negative affect or self-rated health. These measures explained at most 55% of the variance in subjective ageing. Thus, SA, ATOA and AgeCog all capture something more than either just general positive/negative dispositions or well-being. This finding underlines that research on mortality and health in old age can benefit from including subjective ageing over and beyond depressive symptoms and negative affect, given its “added value” for predicting developmental outcomes in later life (cf. Westerhof et al. 2014).

The fact that all three subjective ageing measures were significantly correlated substantiates their convergent validity. Correlations between ATOA and three of the four AgeCog scales (AgeCog Physical Losses, AgeCog Social Losses, AgeCog Ongoing Development) were substantial, all exceeding *r* > .50. Findings with regard to the convergent validity of AgeCog Self-Knowledge were somewhat less conclusive: AgeCog Self-Knowledge was related to both ATOA and AgeCog Ongoing Development, but less so to AgeCog Social Losses and AgeCog Physical Losses. This might, however, be a matter of domain-specificity, given that apart from AgeCog Ongoing Development, all other three AgeCog scales showed distinct relationship profiles with dispositional and well-being variables (see below). It is also noteworthy that inter-relations among the four AgeCog scales did not exceed |.63|. In contrast to ATOA which entails a *global* assessment of one’s own ageing process, the AgeCog scales were designed to reflect the multidimensional nature of subjective ageing (Diehl et al. 2014; Kornadt and Rothermund 2012; Steverink et al. 2001).

The correlations between SA and the other subjective ageing measures were low and low-to-moderate (|.09| to |.30|). This result is in line with theoretical conceptualizations of subjective ageing, which treat SA and self-perceptions of ageing (as captured by measures like ATOA and AgeCog) as related, but also distinct constructs (Diehl et al. 2014; Wurm et al. 2017). We found that the included dispositional and well-being variables explained only 10% of variance in SA. The low correlation with other subjective ageing measures and the low proportion of explained variance resonate with the ongoing debate about what SA actually measures (Gendron et al. 2018; Zacher and Rudolph 2018). SA can be regarded as a highly aggregated construct that is presumably based on personal experiences of ageing in many different domains (e.g. biological, social, psychological) as well as on environmental and social cues about ageing (cf. Kotter-Grühn et al. 2016; Montepare 2009). Recent studies have identified several psychological and biological predictors of SA (Bellingtier and Neupert 2019; Spuling et al. 2013; Stephan et al. 2015, 2018a), but a theoretical framework that sheds light on the nature of this intriguing construct is still needed. We found that, of the five studied dispositional and well-being variables, self-rated health explained the highest (though still small) proportion of variance in SA. People therefore appear to assess how old they feel based at least in part on their physical functioning (see also Stephan et al. 2019).

Of all three studied subjective ageing measures, ATOA most highly correlated with the general dispositional and well-being variables. The relationships between ATOA and general dispositions and well-being might mirror the history of the ATOA subscale, since the PGCMS was originally designed to measure subjective well-being in later life (Liang and Bollen 1983). Also, this result suggests that individuals’ *global* assessments of their ageing process are less unique than multidimensional assessments; the latter of which appear to be less related to a general dispositional tendency to see the future in a positive light. Thus, caution is warranted when drawing conclusions about the predictive power of subjective ageing when using the ATOA measure without statistically controlling for general dispositional and well-being variables such as optimism. At older ages, however, optimism might be more situated than dispositional. Multicollinearity must therefore be ruled out before optimism can be included as a statistical control.^1^

The AgeCog scales—particularly AgeCog Ongoing Development—seem to be somewhat more related to dispositional and well-being measures than SA, but less so than ATOA. The dispositional and well-being measures explained almost half of the variance in AgeCog Ongoing Development, but just one-fifth to one-third of the variance in other three AgeCog scales. It therefore seems that AgeCog Physical Losses, Social Losses and Self-Knowledge are more specific than AgeCog Ongoing Development. Unlike the items in the other AgeCog scales, which refer to the status quo, the items of the Ongoing Development scale (like the optimism items) all refer to the future, using verbs such as “become”, “retain”, “continue”. Potentially, ageing expectations are more highly related to general dispositions directed to positive future outcomes than are evaluations of past and present ageing (cf. Klusmann et al. 2019a). This idea is substantiated by the high correlation of AgeCog Ongoing Development with optimism (*r* = .61). AgeCog Ongoing Development was additionally highly related to the other general disposition considered in the present study, namely self-efficacy (*r* = .52). This strong association is in line with Lachman’s (2006) findings on the benefits of perceived control over ageing.

The exclusive relationship profile of AgeCog Physical Losses with self-rated health and, similarly, that of AgeCog Social Losses with negative affect and depressive symptoms argue in favour of the notion that multidimensionality can and must be regarded for the study of subjective ageing. Several empirical studies lend support to the notion of a match in content between dimension-specific subjective ageing aspects and outcomes (e.g. health-specific subjective ageing and health outcomes; social-specific subjective ageing and social outcomes) as evidenced by exclusive or stronger relationships (e.g. Klusmann et al. 2019b; Levy and Leifheit-Limson 2009).

### Limitations

Our study has a number of strengths, above all the use of three popular subjective ageing measures, a range of well-established dispositional and well-being variables, a large, heterogeneous sample of middle-aged and older adults, and a comprehensive analytic strategy. The reflection on some limitations, however, might help to guide further research. First of all, future studies should consider additional subjective ageing measures. Studying domain-specific subjective age (e.g. Kornadt et al. 2017), other self-perceptions of ageing measures such as the Expectations Regarding Aging scale (Sarkisian et al. 2002, 2005) or novel dynamic process-oriented approaches such as Awareness of Age-related Change (Brothers et al. 2017) would allow for further insights into the validity of different subjective ageing measures and the inter-relations between concepts.

Furthermore, objective measures of positive or negative functioning (e.g. health, mortality), age-diverse and intercultural samples, or even a sophisticated multi-trait, multimethod approach would provide additional evidence for the validity and uniqueness of the subjective ageing construct as a whole. Ideally, future subjective ageing measures should be developed on the basis of a strong theoretical rationale that explicitly elaborates on how the measure is similar to as well as different from other measures.

## Conclusion

In sum, we conclude that how people experience, evaluate and anticipate their own old age and ageing *is something unique* and clearly represents something more than their general dispositions or well-being. At the same time, our results also highlight the conceptual and empirical distinctions between different subjective ageing measures. Currently, the literature offers little guidance about how researchers can determine which measure best suits their objectives. Based on our results, we advise researchers to be mindful of the differences between subjective ageing constructs and measures. We found that SA was least similar to the other constructs used in our study. SA thus does *not* represent a reasonable substitute for the longer ATOA or AgeCog scales. Furthermore, ATOA was shown to be less distinct from general dispositional and well-being variables than the multidimensional AgeCog scales. Given its high correlation with optimism, we recommend that researchers interested in using ATOA as a key variable of interest also assess optimism to rule out possible overlap. Finally, our study highlights the merit of multidimensional and domain-specific conceptualizations of subjective ageing which are necessary for understanding distinct subjective ageing processes and dynamics.

## Electronic supplementary material

Below is the link to the electronic supplementary material.

Supplementary material 1 (DOCX 17 kb)

## Footnotes

1 We thank an anonymous reviewer for this suggestion.
