# Supplementary material for: The uniqueness of subjective ageing: convergent and discriminant validity
Source: Eur J Ageing. 2019 Oct 9;17(4):445–55. doi: 10.1007/s10433-019-00529-7 (PMC7752926; doi:10.1007/s10433-019-00529-7)
Supplement: Supplementary file 1 — Supplementary material 1 (DOCX 17 kb) [file 10433_2019_529_MOESM1_ESM.docx]

**Table 2 with bivariate raw correlations and corrected correlations for scale reliability where applicable between subjective ageing, general dispositional and well-being measures for Article “The Uniqueness of Subjective Ageing: Convergent and Discriminant Validity”**

Spuling, Klusmann, Bowen, Kornadt & Kessler

**Table 2**

*Bivariate Correlations (Raw and Corrected for Scale Reliabilities Where Applicable) between Subjective Ageing Measures and General Dispositional and Well-being Variables for the Total Sample*

|  | Cronbach’s Alpha | 1 | 2 | 3 | 4 | 5 | 6 | 7 | 8 | 9 | 10 |
| --- | --- | --- | --- | --- | --- | --- | --- | --- | --- | --- | --- |
| **1 SA** |  |  |  |  |  |  |  |  |  |  |  |
| **2 ATOA** | .74 | -.30 |  |  |  |  |  |  |  |  |  |
| **3 AgeCog  Physical Losses** | .76 | .23 | -.53 |  |  |  |  |  |  |  |  |
|  |  |  | *-.70* |  |  |  |  |  |  |  |  |
| **4 AgeCog  Social Losses** | .71 | .17 | -.61 | .44 |  |  |  |  |  |  |  |
|  |  |  | *-.84* | *.60* |  |  |  |  |  |  |  |
| **5 AgeCog  Ongoing Development** | .78 | -.22 | .60 | -.40 | -.43 |  |  |  |  |  |  |
|  |  |  | *.80* | *-.52* | *-.58* |  |  |  |  |  |  |
| **6 AgeCog  Self-Knowledge** | .57 | -.09 | .35 | -.11 | -.26 | .42 |  |  |  |  |  |
|  |  |  | *.53* | *-.16* | *-.40* | *.63* |  |  |  |  |  |
| **7 Optimism** | .83 | -.23 | .69 | -.44 | -.51 | .61 | .37 |  |  |  |  |
|  |  |  | *.88* | *-.54* | *-.67* | *.76* | *.53* |  |  |  |  |
| **8 Self-Efficacy** | .74 | -.18 | .47 | -.29 | -.38 | .52 | .42 | .57 |  |  |  |
|  |  |  | *.63* | *-.39* | *-.53* | *.68* | *.64* | *.73* |  |  |  |
| **9 Depressive Symptoms** | .86 | .21 | -.45 | .28 | .36 | -.28 | -.15 | -.44 | -.32 |  |  |
|  |  |  | *-.56* | *.35* | *.45* | *-.34* | *-.21* | *-.52* | *-.40* |  |  |
| **10 Negative Affect** | .85 | .12 | -.37 | .24 | .41 | -.20 | -.19 | -.40 | -.39 | .48 |  |
|  |  |  | *-.47* | *.30* | *.52* | *-.25* | *-.27* | *-.47* | *-.49* | *.56* |  |
| **11 Self-Rated Health** |  | -.27 | .45 | -.40 | -.24 | .33 | .14 | .38 | .26 | -.46 | -.23 |

*Notes*. All coefficients significant at *p* < .01. Correlation corrected for scale reliabilities in italics where applicable. SA = Subjective age; ATOA = Attitude toward own ageing subscale, AgeCog = Ageing cognitions scales
